# Multiple sclerosis and legal capacity

**DOI:** 10.1002/alz.084303

**Published:** 2025-01-03

**Authors:** Panagiota Voskou

**Affiliations:** ^1^ University of Athens, Athens Greece

## Abstract

**Background:**

Cognitive deficits may occur in about 60% of patients with multiple sclerosis (MS) and executive functions, working memory and new‐learning are commonly impaired. Nevertheless, there is limited research regarding decisional capacity in MS. Financial and treatment‐decision capacity are complex activities of daily living.

**Method:**

A literature review in the Pubmed database has been made, using the keywords: multiple sclerosis, legal capacity, decision‐making.

**Result:**

Overall financial capacity (FC) and more complex financial domains, i.e. managing bank accounts, are significantly impaired in progressive MS, with mental flexibility and working memory mostly involved. Short‐term verbal memory and basic arithmetic ability are found to be major neurocognitive predictors of FC in progressive MS. Ability to understand treatment and clinical research is commonly impaired in MS, i.e. misunderstanding treatment details due to diminished new‐learning or executive functions or difficulty comprehending treatment risks and benefits. Recognition cueing is found to be helpful for robust informed consent from MS patients. Depressive distress is also common in MS and this corresponds with poor reasoning and, subsequently, decisional incapacity.

**Conclusion:**

More studies should be conducted about the capacity for informed consent and financial tasks in MS, as well as which neurocognitive functions are related to specific components of medical decision‐making. The primary goal should be the protection of rights and autonomy in MS. Depression and its neurocognitive correlates in MS should be also examined in future research.

